# A novel antibacterial strategy: histone and antimicrobial peptide synergy

**DOI:** 10.15698/mic2020.11.736

**Published:** 2020-10-08

**Authors:** Leora Duong, Steven P. Gross, Albert Siryaporn

**Affiliations:** 1 Department of Molecular Biology & Biochemistry, UC Irvine, Irvine, CA 92697, USA.; 2 Department of Developmental and Cell Biology, UC Irvine, Irvine, CA 92697, USA.; 3 Department of Physics & Astronomy, UC Irvine, Irvine, CA 92697, USA.

**Keywords:** antibiotics, membrane pore stabilization, chromosome reorganization, innate immunity

## Abstract

The rate at which antibiotics are discovered and developed has stagnated; meanwhile, antibacterial resistance continually increases and leads to a plethora of untreatable and deadly infections worldwide. Therefore, there is a critical need to develop new antimicrobial strategies to combat this alarming reality. One approach is to understand natural antimicrobial defense mechanisms that higher-level organisms employ in order to kill bacteria, potentially leading to novel antibiotic therapeutic approaches. Mammalian histones have long been reported to have antibiotic activity, with the first observation of their antibacterial properties reported in 1942. However, there have been doubts about whether histones could truly have any such role in the animal, predominantly based on two issues: they are found in the nucleus (so are not in a position to encounter bacteria), and their antibiotic activity *in vitro* has been relatively weak in physiological conditions. More recent studies have addressed both sets of concerns. Histones are released from cells as part of neutrophil extracellular traps (NETs) and are thus able to encounter extracellular bacteria. Histones are also present intracellularly in the cytoplasm attached to lipid droplets, positioning them to encounter cytosolic bacteria. Our recent work (Doolin et al., 2020, Nat Commun), which is discussed here, shows that histones have synergistic antimicrobial activities when they are paired with antimicrobial peptides (AMPs), which form pores in bacterial membranes and co-localize with histones in NETs. The work demonstrates that histones enhance AMP-mediated pores, impair bacterial membrane recovery, depolarize the bacterial proton gradient, and enter the bacterial cytoplasm, where they restructure the chromosome and inhibit transcription. Here, we examine potential mechanisms that are responsible for these outcomes.

At physiological ionic concentrations *in vitro*, histones by themselves have little impact on bacterial growth. The work sought to address an outstanding question in the field: why do histones exhibit relatively weak antimicrobial activity *in vitro* despite having clear antimicrobial activity *in vivo*? The work provides a potential answer to this by recognizing that *in vivo*, histones are not by themselves but are instead surrounded by other antimicrobial molecules. In both NETs and lipid droplets, histones co-localize with pore-forming AMPs. By themselves, pore-forming AMPs, including LL-37, magainin-2, and polymyxin B, have inhibitory effects on bacterial growth. However, when these AMPs are paired with histones, the inhibitory effect is amplified and synergistic. That is, the combined inhibitory effect of AMPs and histones on bacterial growth is greater than the sum of the individual effects.

Such synergistic antimicrobial interactions could improve the efficacy of new and existing antimicrobial agents. How does the synergy between histones and AMPs arise? The study focuses on the activity of histone H2A, one of the four core histone proteins, and the cathelicidin-derived AMP LL-37. Both H2A and LL-37 are comparable in size (14 and 18 kDa, respectively), are cationic, contain a high proportion of hydrophobic amino acids, and possess the ability to form alpha helices. The overlapping similarities suggest that both molecules have similar impacts on bacterial physiology. The work reveals, however, that the molecules perform distinct functions and that the complementarity of the mechanisms give rise to synergistic antimicrobial activity. For example, whereas LL-37 alone increases membrane permeability, H2A alone has little impact. While H2A binds DNA and condenses chromosomes, LL-37 has no such function. When LL-37 and H2A are paired together, these functions combine and produce irreparable damage by targeting two sites: the bacterial membrane and the cytoplasm. Other AMPs, including magainin-2 and polymyxin-B, exhibit similar antimicrobial synergies with H2A. How exactly does this enhancement by histones work?

## HISTONES ENHANCE MEMBRANE PERMEATION

**Figure 1:**
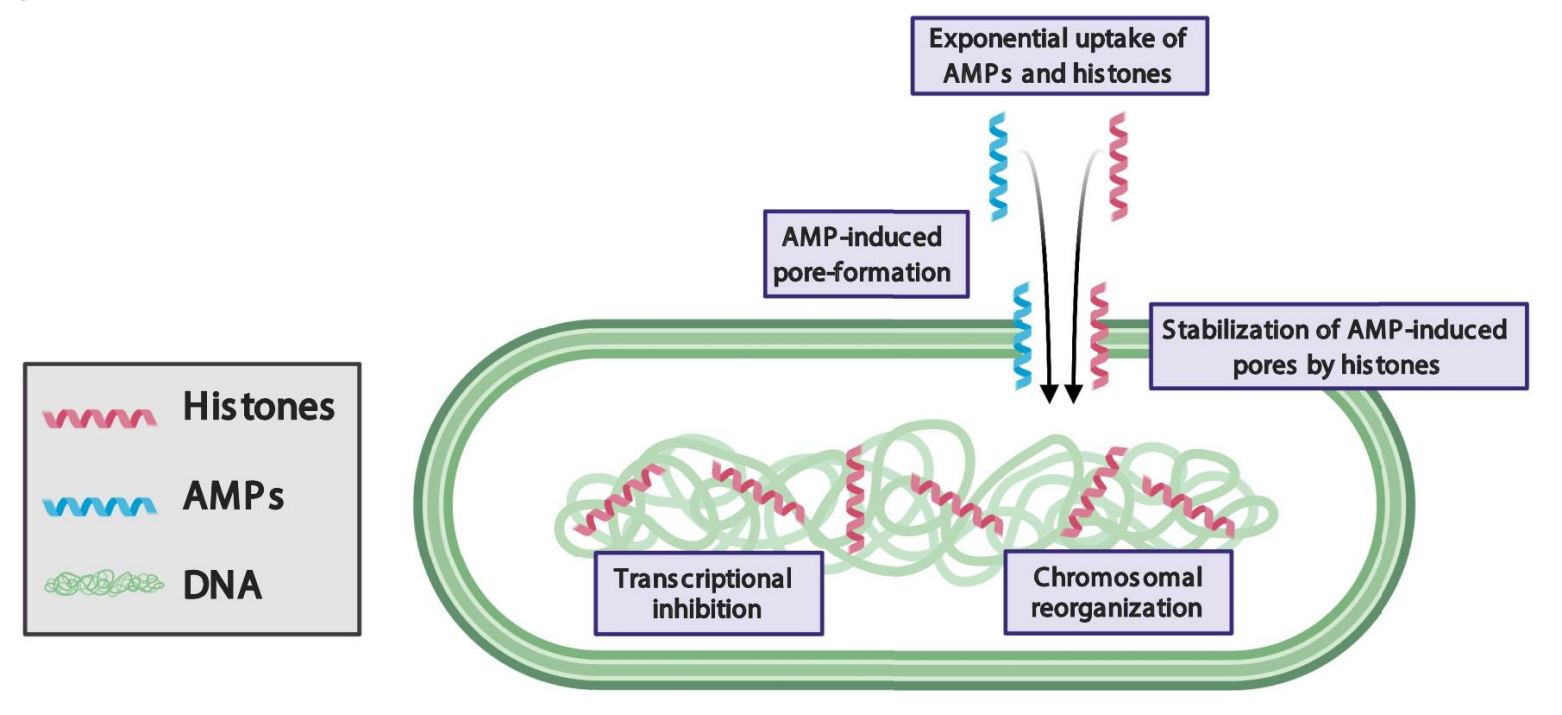
FIGURE 1: Model of antibacterial synergy between mammalian histones and pore-forming antimicrobial peptides (AMPs). AMPs induce pore formation in bacterial membranes, which facilitates the entry of histones. Histones stabilize AMP-induced pores, reorganize the chromosome, and inhibit transcription.

LL-37 has previously been reported to inhibit growth by forming pores in bacterial membranes. Our study demonstrates that bacteria recover from the pore-forming effects of LL-37 after the molecule is removed. Remarkably, when bacteria are treated with both LL-37 and H2A, the pore-forming effects to the membrane are persistent and irrecoverable. The persistence of membrane pores facilitates the leakage of components out of the cytoplasm and enables the entry of additional histones and LL-37 into the cell. This suggests that H2A stabilizes LL-37-induced pores (**Figure 1**).

How histones could stabilize AMP-induced pores is unknown. The stabilization could arise through indirect interactions between histone and AMP mechanisms in which histones may alter the chemical or physical properties of membranes. Our fluorescence imaging shows that histones significantly increase the recruitment of LL-37 to the membrane, where LL-37 could create greater membrane stress and a greater number of or larger pores. In addition, our scanning electron microscopy images indicate that histones cause membrane surfaces to appear rough, which suggests that histones alter the mechanical properties of the membrane. A significant change increase in membrane tension due to histones could facilitate pore formation by AMPs and likewise, would increase the barriers against closing any AMP-induced pores. Alternatively, histones could stabilize pores through direct interaction with AMPs. Such interactions could result in the creation of new complexes that widen AMP-induced pores, hold open pores for longer periods of time, obstruct membrane repair, inhibit drug efflux pumps, or a combination of these mechanisms.

The data suggest a combination of these mechanisms contribute to increasing the pore forming action of AMPs, which impedes the ability of bacteria to recover. Future experiments that probe the localization and binding of histones and AMPs, measure the binding affinities between histones and AMPs, and probe membrane mechanics will address the extent to which each mechanism is involved. Regardless of the how histones stabilize pores, the impact of pore stabilization is clear: the bacterial membrane proton gradient is destroyed. This gradient is necessary for ATP energy production and without it, bacteria cannot carry out essential cellular functions.

## CHROMOSOMAL REORGANIZATION

In eukaryotes, histones bind to and regulate chromosomes. In bacteria, histone-like nucleoid structuring (H-NS) proteins regulate chromosomal structure and transcription. However, when membrane pores are formed, the facilitated entry of eukaryotic histones into the bacterial cytoplasm may cause dysregulation of functions associated with H-NS. Histone H2A binds to bacterial DNA, reorganizes the structure of bacterial chromosomes, and inhibits transcription (**Figure 1**). Thus, in addition to the damaging effects of histones at the membrane, histones attack bacteria via intracellular targets. As a result, bacteria are unable to maintain the expression of genes that are essential for growth and duplication and are unable to express genes that could repair membrane pores, such as those that produce lipid components. Transcriptional inhibition also suppresses genes that could provide resistance to AMPs and histones, including drug efflux pumps and membrane charge-modifying components. It remains to be explored the extent to which bacterial defense mechanisms are suppressed by the histones. Many questions remain about the effects of histones on bacterial chromosomes, including how they affect the function of H-NS proteins, how the accessibility to transcription sites by RNA polymerase is affected, and the existence of high affinity histone-binding sites in the chromosome.

## SYNERGY THROUGH A POSITIVE FEEDBACK LOOP

A notable effect of histones and AMPs is their ability to increase the uptake of the partner molecule into bacteria: H2A increases the uptake of LL-37 and LL-37 increases the uptake of H2A. These dynamics give rise to a positive feedback loop that exponentially increases the intracellular concentration of both molecules. The individual use of histones or AMPs results in little uptake and relatively weak antimicrobial activity. In contrast, their combination gives rise to a synergistic mechanism in which both drugs are taken up into the cytoplasm at significantly greater concentrations, resulting in greater antimicrobial activity. These results suggest that the incorporation of a positive feedback loop into the design strategy of antimicrobial drugs could greatly increase their efficacy.

## CONCLUDING REMARKS

The dual treatment strategy described in this work can be used to invigorate current antibiotics that are under development. Possible therapeutic strategies include manipulating the release of histones and AMPs in NETs or lipid droplets to regulate levels of free histones to inhibit bacterial growth. On the other hand, safer and more efficient analogs of histones and AMPs can also be developed to treat an array of infections. Topical treatments can also be made from the synergistic treatments, where histone toxicity is less of a concern. The ability of the combined antimicrobial treatment to act via multiple distinct modes of action potentially increases the barrier for the development of bacterial resistance to the treatment. The targeting of both bacterial membranes and intracellular functions positions the histones and AMP dual treatment as a potentially effective antimicrobial strategy against both Gram-negative and Gram-positive bacteria. This antimicrobial approach provides a refreshing perspective toward the revitalization of current antibiotic development strategies.

